# Supplementary material for: Proteins with proximal-distal asymmetries in axoneme localisation control flagellum beat frequency
Source: Nat Commun. 2025 Apr 4;16:3237. doi: 10.1038/s41467-025-58405-1 (PMC11971395; doi:10.1038/s41467-025-58405-1)
Supplement: Supplementary file 1 — Supplementary Information [file 41467_2025_58405_MOESM1_ESM.pdf]

Supplemental Material

Protein name	Gene ID	Position	Electron density change upon deletion	DC-dependent	ODA-dependent	Flagellum length	Swim speed	Tip-to-base beat incidence	Base-to-tip beat incidence	Tip-to-base beat frequency
pDC1	LmxM.10.0960	Proximal	Reduced near ODA (low confidence)	Yes	No	No change	Slow	Normal	Normal	Bimodal
pDC2	LmxM.06.1040	Proximal	Reduced near ODA (low confidence)	N/A	No	No change	Normal	Less	More	Bimodal
FLAM6	LmxM.08_29.1700	Proximal	Reduced near ODA	Yes	Yes	No change	Normal	Normal	Normal	Increased
pDC4	LmxM.29.0240	Proximal	Reduced near ODA	Yes	No	No change	Normal	Less	More	Normal
PDAP1	LmxM.31.2530	Proximal	Reduced near ODA	Yes	No	No change	Normal	Normal	Normal	Increased
SPA2	LmxM.32.0390	Proximal	None	No	No	No change	Slow	Normal	Normal	Bimodal
SPA1	LmxM.36.5300	Proximal	None	No	Yes	No change	Normal	Normal	Normal	Increased
ARL13B	LmxM.36.0820	Proximal	None	No	Yes	Short	N/D	N/D	N/D	N/D
dDC1	LmxM.15.0540	Distal	Reduced ODA	Yes	No	No change	Very slow	Less	More	Decreased
dDC2	LmxM.31,2900	Distal	Reduced ODA	Yes	No	No change	Very slow	Less	More	Decreased
LC4-like	LmxM.01.0620	Distal	Reduced near ODA	Yes	Yes	No change	Fast	Less	Normal	Increased
dDC4	LmxM.30.0090	Distal	None	Yes	Yes	No change	Normal	Less	Normal	Normal
PDEB2	LmxM.15.1480	Distal	None	No	No	No change	Normal	Less	More	Normal
PDEB1	LmxM.15.1481	Distal	None	No	No	No change	Normal	Less	More	Increased

Table S1. Qualitative summary of protein localisation and deletion mutant phenotypes in *L. mexicana*.

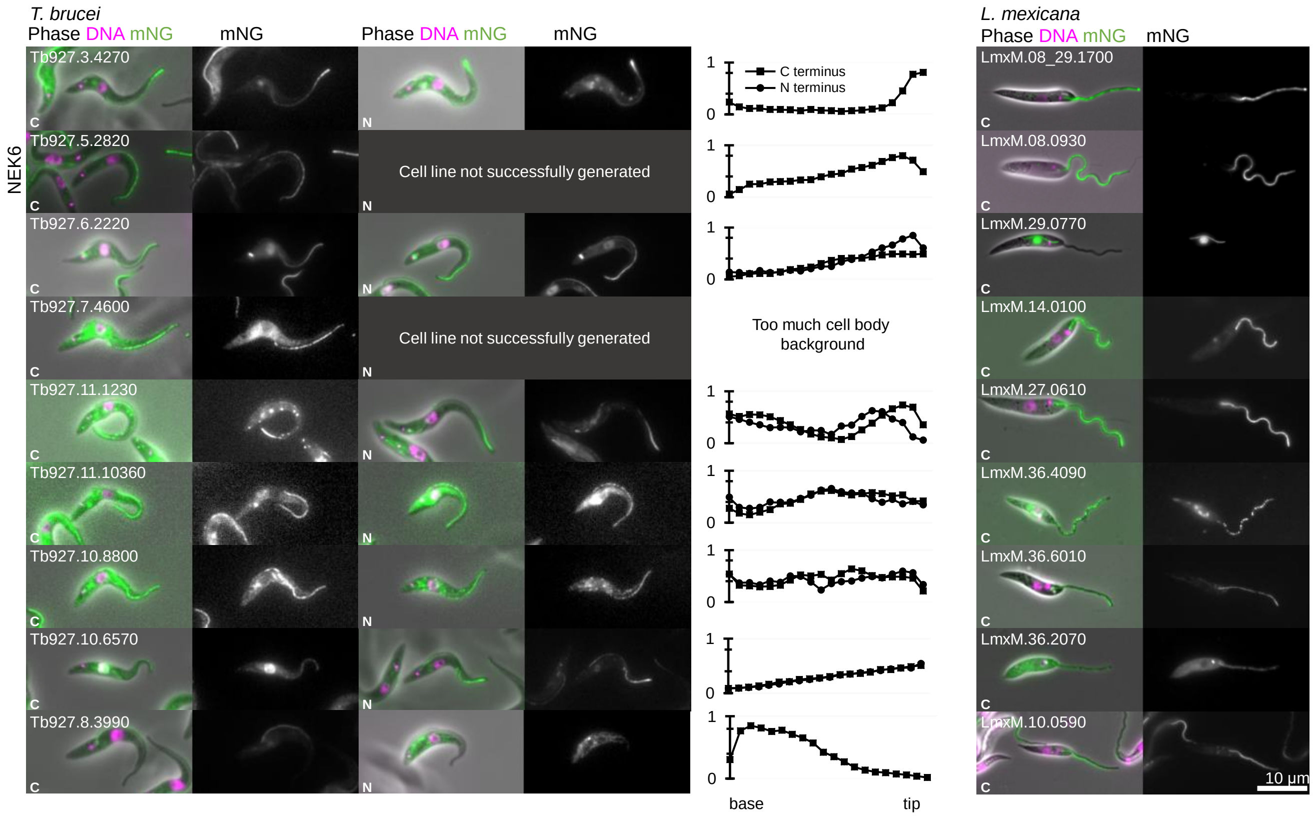

Figure S1. Proximal or distal axoneme-specific proteins in *T. brucei* where the *L. mexicana* ortholog did not have a proximal or distal-specific localisation. The first column shows widefield epifluorescence micrographs of mNG fluorescence at the C terminus in distal proteins in *T. brucei*, then the second column shows widefield epifluorescence micrographs of mNG fluorescence at the N terminus in distal proteins in *T. brucei*. Phase contrast (grey), DNA (Hoechst 33342, magenta) and mNG (green) overlay and mNG fluorescence are shown. In the third column, graphs representing the mNG fluorescence signal intensity along the axoneme, from the base to the tip. Data points represent the mean of n = 15 axonemes in 1K1N cells, normalised by maximum signal intensity per cell. The fourth column shows widefield epifluorescence of endogenous tagging at the C terminus of the *L. mexicana* ortholog. Source data are provided in the Source Data file.

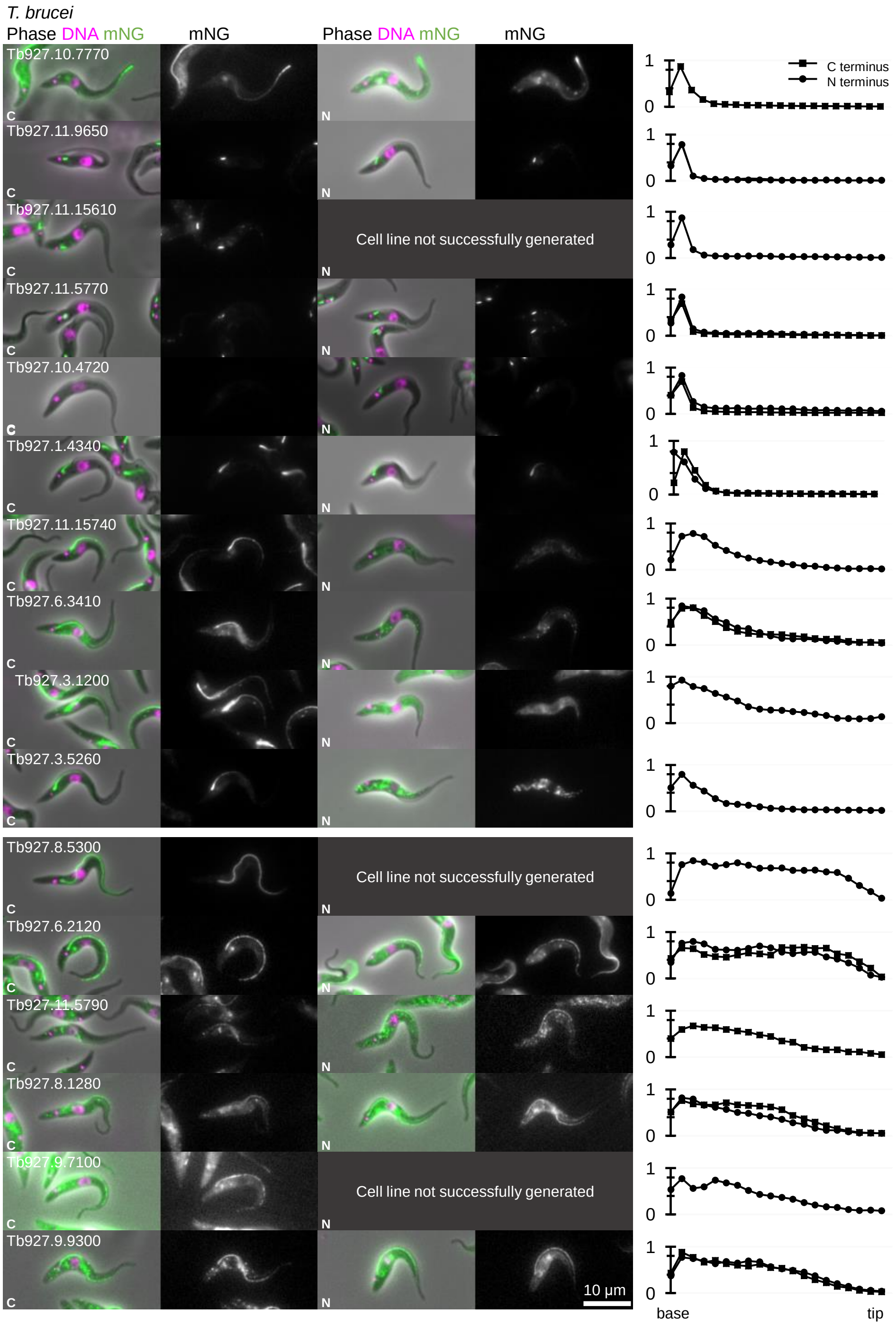

Figure S2. Proximal axoneme-specific proteins in *T. brucei* which lack a detectable *L. mexicana* ortholog. The first column shows widefield epifluorescence micrographs of mNG fluorescence at the C terminus in distal proteins in *T. brucei*, then the second column shows widefield epifluorescence micrographs of mNG fluorescence at the N terminus in distal proteins in *T. brucei*. Phase contrast (grey), DNA (Hoechst 33342, magenta) and mNG (green) overlay and mNG fluorescence are shown. In the third column, graphs representing the mNG fluorescence signal intensity along the axoneme, from the base to the tip. Data points represent the mean of *n* = 15 axonemes in 1K1N cells, normalised by maximum signal intensity per cell. Source data are provided in the Source Data file.

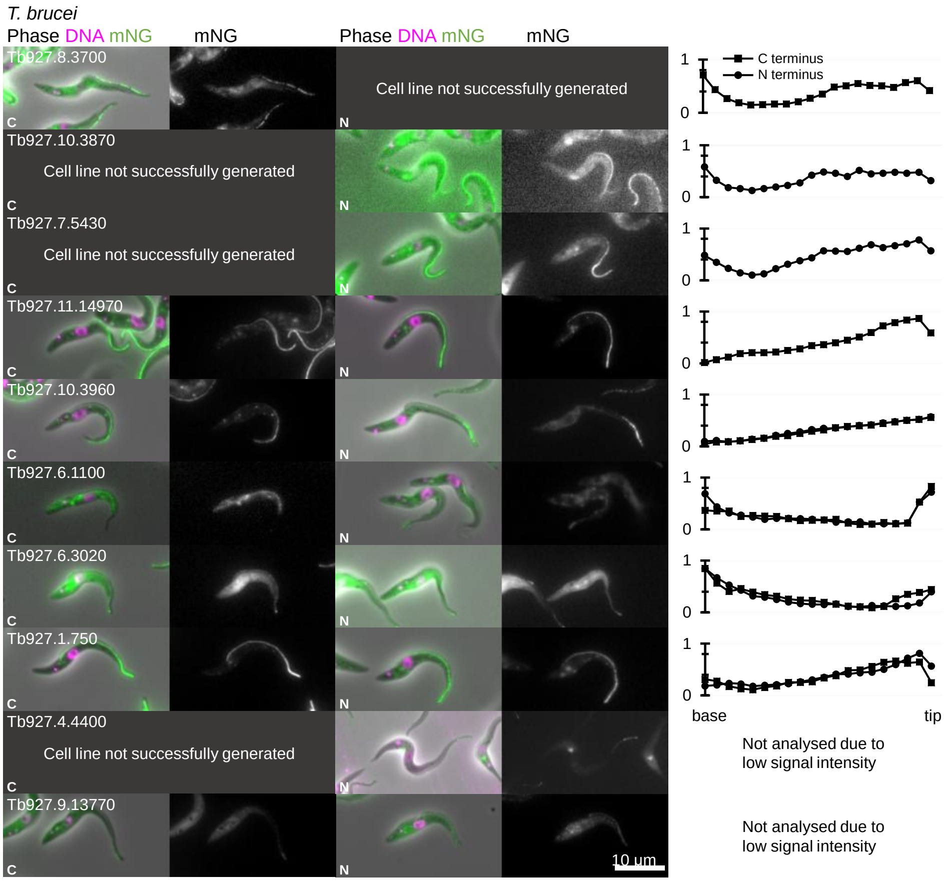

Figure S3. Distal axoneme-specific proteins in *T. brucei* which lack a detectable *L. mexicana* ortholog. The first column shows widefield epifluorescence micrographs of mNG fluorescence at the C terminus in distal proteins in *T. brucei*, then the second column shows widefield epifluorescence micrographs of mNG fluorescence at the N terminus in distal proteins in *T. brucei*. Phase contrast (grey), DNA (Hoechst 33342, magenta) and mNG (green) overlay and mNG fluorescence are shown. In the third column, graphs representing the mNG fluorescence signal intensity along the axoneme, from the base to the tip. Data points represent the mean of *n* = 15 axonemes in 1K1N cells, normalised by maximum signal intensity per cell. Source Data are provided in the Source Data file.

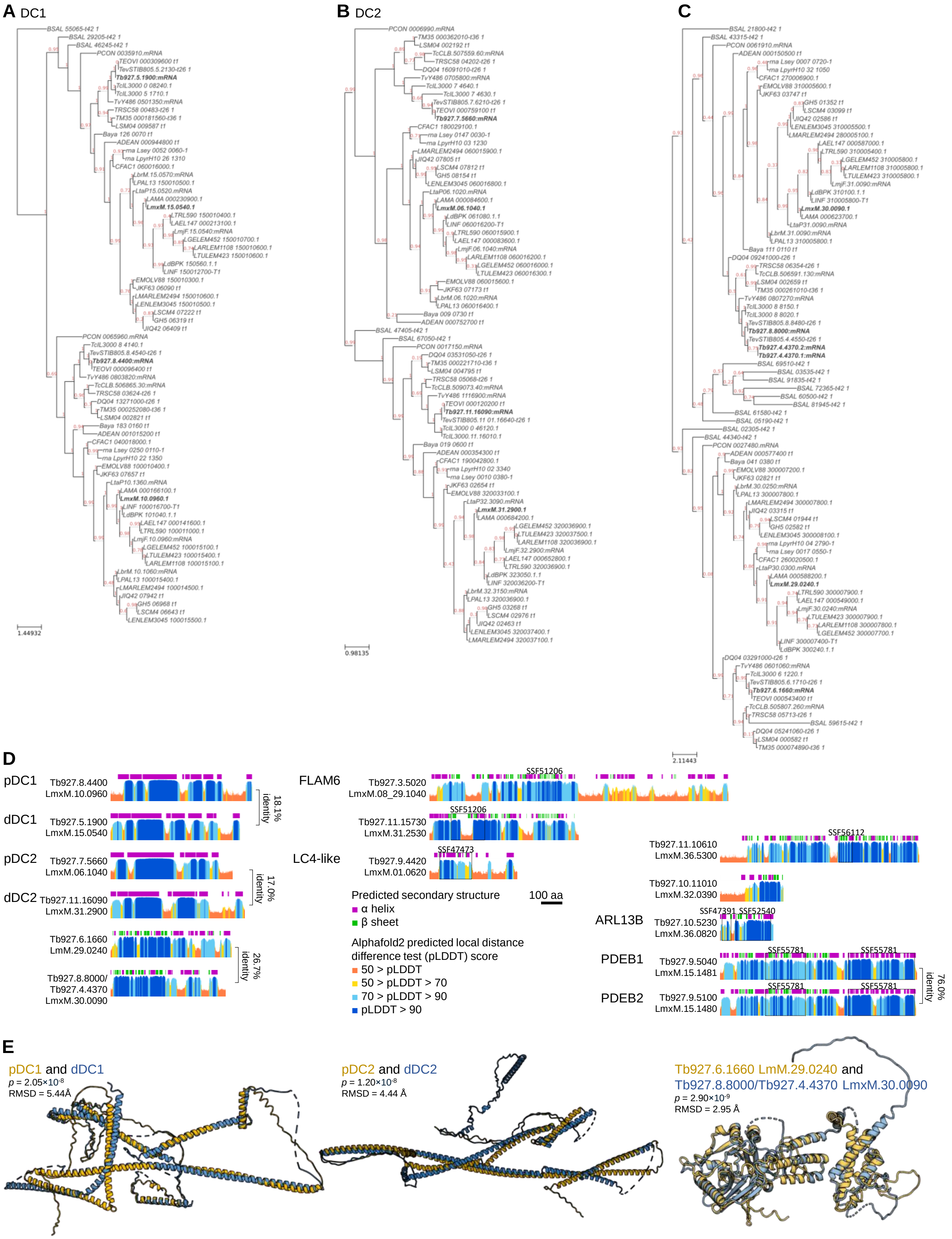

A DC1
B DC2
C
1.44932
0.98135
2.11443
D
pDC1 Tb927.8.4400 LmxM.10.0960
dDC1 Tb927.5.1900 LmxM.15.0540
18.1% identity
pDC2 Tb927.7.5660 LmxM.06.1040
dDC2 Tb927.11.16090 LmxM.31.2900
17.0% identity
Tb927.6.1660 LmM.29.0240
Tb927.8.8000/ Tb927.4.4370 LmxM.30.0090
26.7% identity
FLAM6 Tb927.3.5020 LmxM.08_29.1040
SSF51206
Tb927.11.15730 LmxM.31.2530
SSF51206
LC4-like Tb927.9.4420 LmxM.01.0620
SSF47473
100 aa
Predicted secondary structure
α helix
β sheet
Alphafold2 predicted local distance difference test (pLDDT) score
50 > pLDDT
50 > pLDDT > 70
70 > pLDDT > 90
pLDDT > 90
Tb927.11.10610 LmxM.36.5300
SSF56112
Tb927.10.11010 LmxM.32.0390
ARL13B Tb927.10.5230 LmxM.36.0820
SSF47391 SSF52540
PDEB1 Tb927.9.5040 LmxM.15.1481
SSF55781 SSF55781
PDEB2 Tb927.9.5100 LmxM.15.1480
SSF55781 SSF55781
76.0% identity
E
pDC1 and dDC1
p = 2.05×10-8
RMSD = 5.44Å
pDC2 and dDC2
p = 1.20×10-8
RMSD = 4.44 Å
Tb927.6.1660 LmM.29.0240 and
Tb927.8.8000/Tb927.4.4370 LmxM.30.0090
p = 2.90×10-9
RMSD = 2.95 Å

Figure S4. Phylogenetic analysis and structural similarities of DC1, DC2 and novel potential DC component orthologs in kinetoplasitds. A-C. Unrooted maximum likelihood phylogenetic trees constructed from orthologs in reference kinetoplastid species, as listed by TriTrypDB for A DC1, B DC2, C orthologs of Tb927.6.1660, Tb927.4.4370 and Tb927.8.8000, which we ultimately name DC4. Leaf labels are the TriTrypDB protein ID, node labels are χ^2 approximate likelihood ratio test p values. D. Summary of AlphaFold2-predicted protein structure and predicted protein domains for the conserved asymmetrically localised proteins. AlphaFold2 structure confidence is summarised by pLDDT, and secondary structure by α helix and β sheet regions. *L. mexicana* and *T. brucei* proteins are all similar, so for simplicity only data from the *T. brucei* ortholog is plotted, specifically Tb927.8.8000 for the Tb927.4.4370/Tb927.8.8000 paralogous pair. E. The structure of the distal *T. brucei* ortholog is aligned to the proximal, using FATCAT to allow twists to align multiple domains. Statistics shown are root mean square deviation (RMSD) and FATCAT structure similarity *p* value.

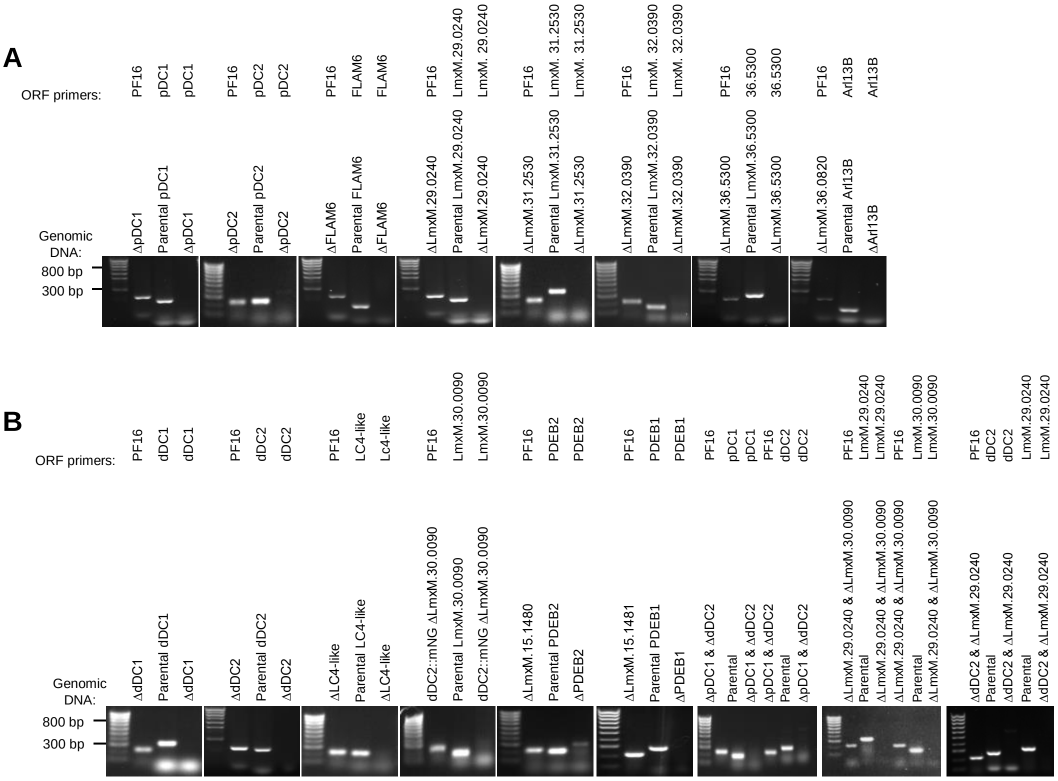

Figure S5. Validation of deletion mutants of proximal and distal-specific axoneme components. Diagnostic PCRs to confirm deletion of both alleles of **A.** proximal and **B.** distal -specific axoneme proteins in *L. mexicana* cell lines. For each, gel electrophoresis of PCR products from genomic DNA (gDNA) are shown. Control PCR product from an unaffected open reading frame (ORF), PF16, are shown to confirm presence of deletion mutant gDNA and PCR products from parental gDNA are shown to confirm that the test primers can amplify the detected ORF.

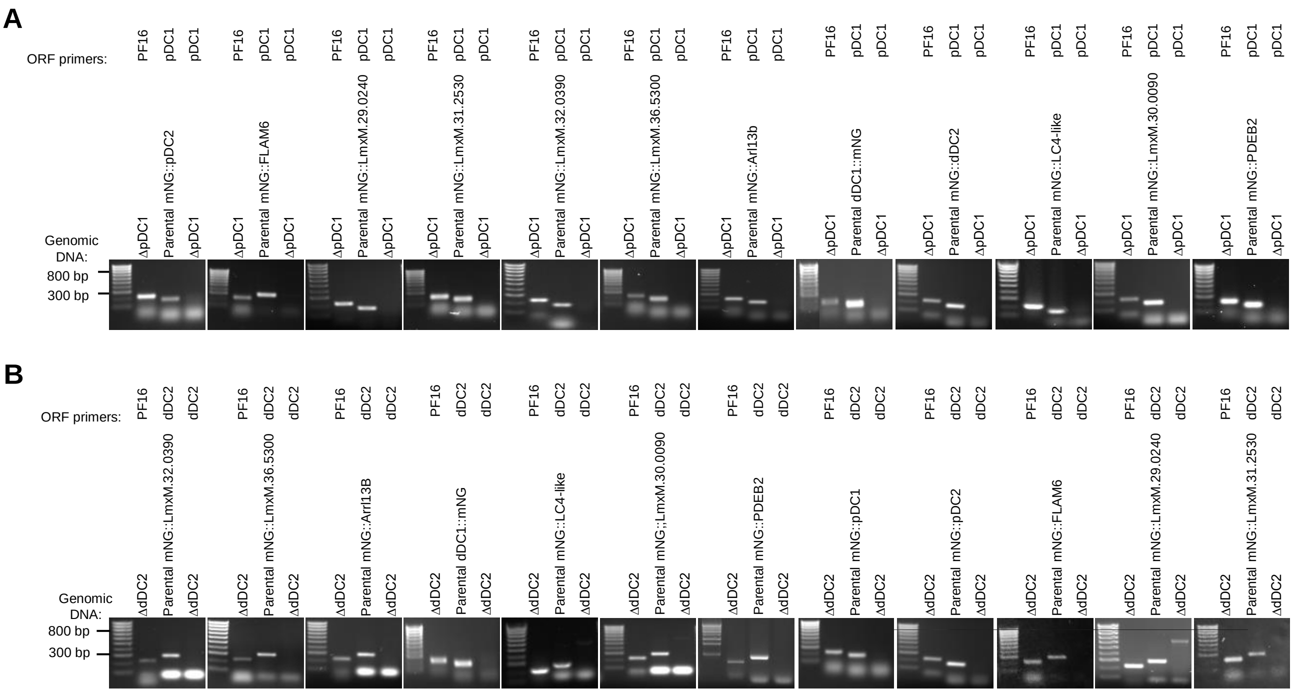

Figure S6. Validation of pDC1 and dDC2 deletion in cell lines with tagged proximal or distal axoneme components. Diagnostic PCRs to confirm deletion of both alleles of **A.** pDC1 and **B.** dDC2 proteins in *L. mexicana* cell lines also expressing a tagged proximal or distal-specific protein. For each, gel electrophoresis of PCR products from genomic DNA (gDNA) are shown. Control PCR product from an unaffected open reading frame (ORF), PF16, are shown to confirm presence of deletion mutant gDNA and PCR products from parental gDNA are shown to confirm that the test primers can amplify the detected ORF.

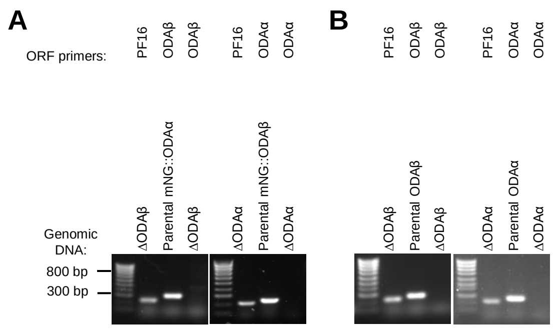

Figure S7. Validation of ODA deletion mutants. Diagnostic PCRs to confirm deletion of both alleles of **A.** ODAβ in cells expressing mNG::ODAα and ODAα in cells expressing tagged mNG::ODAβ and **B.** ODAα and ODAβ proteins in the *L. mexicana* cell lines. For each, gel electrophoresis of PCR products from genomic DNA (gDNA) are shown. Control PCR product from an unaffected open reading frame (ORF), PF16, are shown to confirm presence of deletion mutant gDNA and PCR products from parental gDNA are shown to confirm that the test primers can amplify the detected ORF.

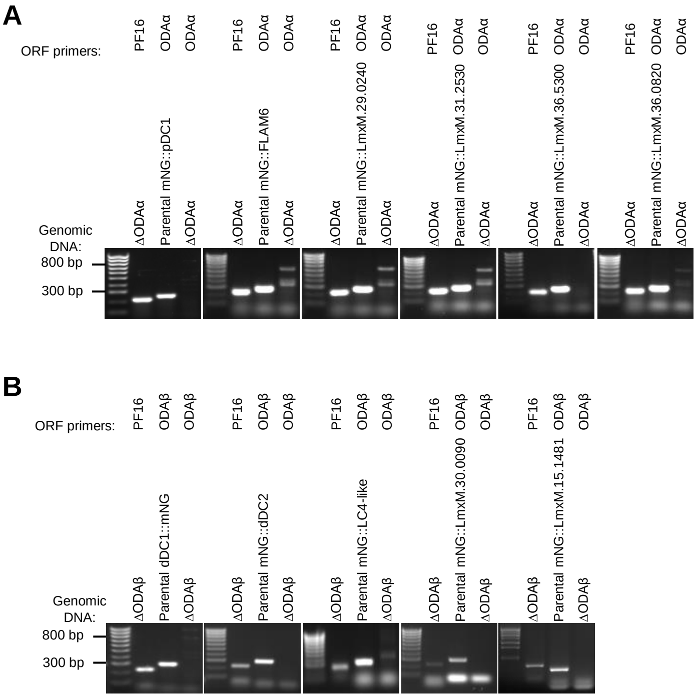

Figure S8. Validation of ODA deletion in cell lines with tagged proximal or distal axoneme components. Diagnostic PCRs to confirm deletion of both alleles of **A.** ODAα in cell lines expressing tagged proximal axoneme proteins and **B.** ODAβ in cell lines with tagged distal proteins. For each, gel electrophoresis of PCR products from genomic DNA (gDNA) are shown. Control PCR product from an unaffected open reading frame (ORF), PF16, are shown to confirm presence of deletion mutant gDNA and PCR products from parental gDNA are shown to confirm that the test primers can amplify the detected ORF.

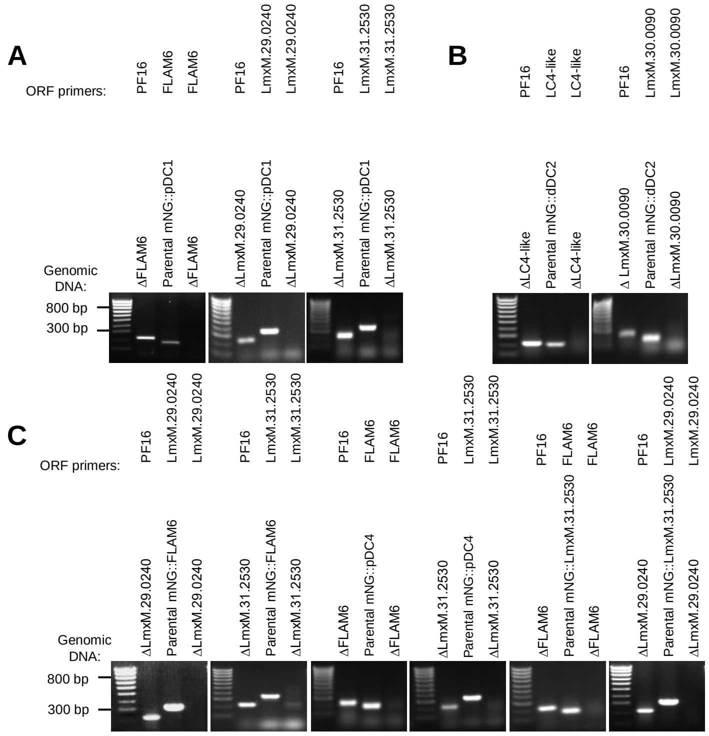

Figure S9. Validation of deletion mutants for combinatorial tagging and deletion of proximal or distal axoneme components. Diagnostic PCRs to confirm deletion of both alleles of **A.** proteins dependent on the pDC in a cell line expressing mNG::pDC1, **B.** proteins dependent on the dDC in a cell line expressing mNG::pDC2 and **C.** combinatorial deletion of proteins dependent on the pDC in cell lines expressing tagged FLAM6, pDC4 orLmxM.31.2530. For each, gel electrophoresis of PCR products from genomic DNA (gDNA) are shown. Control PCR product from an unaffected open reading frame (ORF), PF16, are shown to confirm presence of deletion mutant gDNA and PCR products from parental gDNA are shown to confirm that the test primers can amplify the detected ORF.

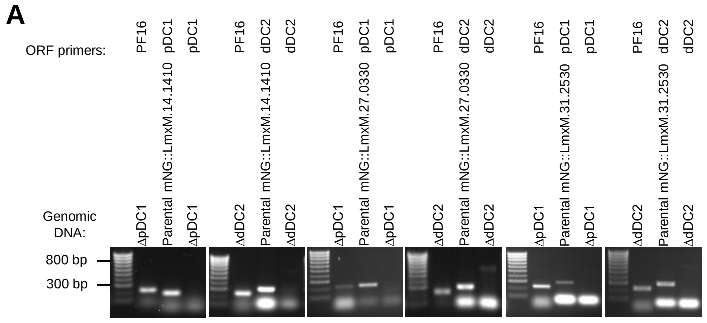

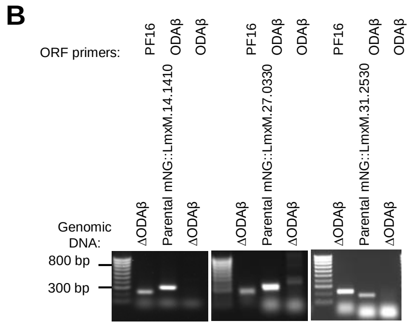

Figure S10. Validation of pDC1, dDC2 and ODAβ deletion mutants in tagged cell lines. Diagnostic PCRs to confirm deletion of both alleles of **A.** pDC1 and dDC2 in cell lines expressing tagged distal proteins and **B.** ODAβ in tagged LmxM.14.1410, LmxM.27.0330 and LmxM.31.2530 in *L. mexicana* cell lines. For each, gel electrophoresis of PCR products from genomic DNA (gDNA) are shown. Control PCR product from an unaffected open reading frame (ORF), PF16, are shown to confirm presence of deletion mutant gDNA and PCR products from parental gDNA are shown to confirm that the test primers can amplify the detected ORF.

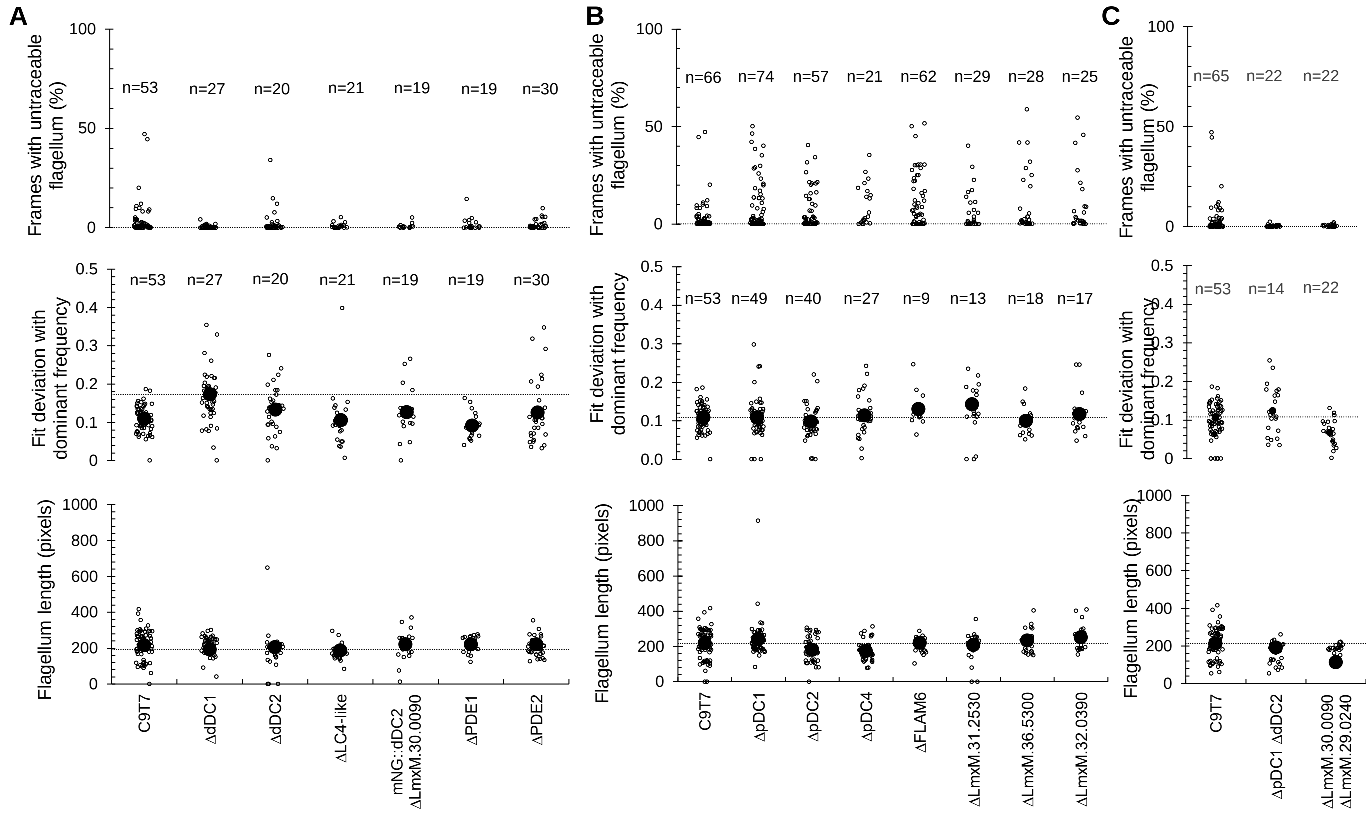

Figure S11. Additional control measures for beat waveform properties for distal and proximal proteins. Graphs representing the percentage of bad frames that are not traceable, the quality of fit using only the dominant frequency and the flagellum length in pixels in A. distal, B. proximal proteins and C. double deletion mutants. Open circles represent each cell, solid circles represent the mean. *n* indicates the number of analysed cells. Source data are provided as a Source Data file.

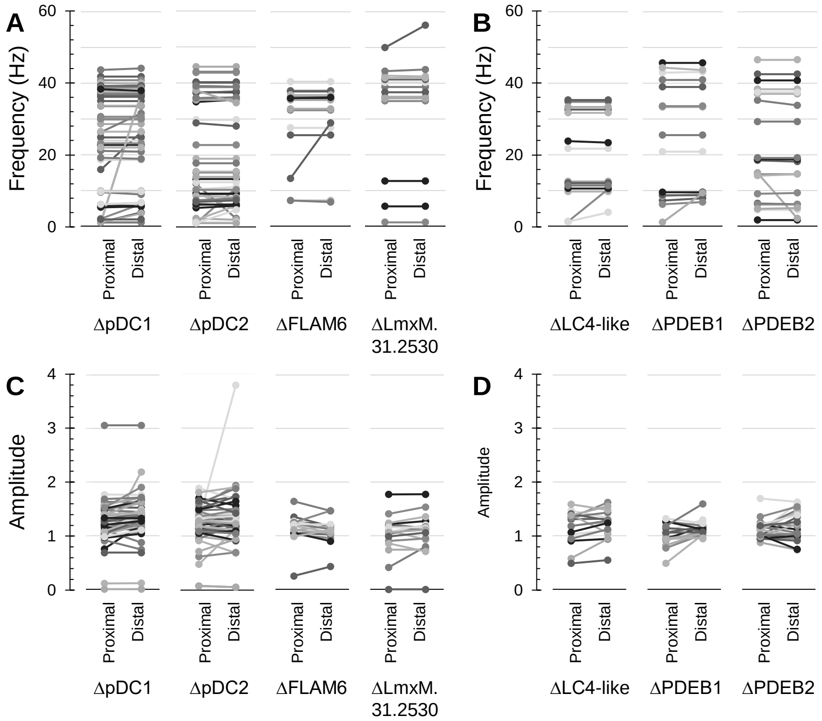

Figure S12. Comparison of dominant beat frequency and amplitude measured from the proximal or distal region of individual flagella, for deletion mutants with a detectably aberrant beat from the whole flagellum. Every line in each graph represents a single flagellum, with the data points corresponding to beat frequency in the proximal and distal flagella A-C. For deletion mutants of proximal proteins, all with DC-dependent localisations: ΔpDC1, ΔpDC2, ΔFLAM6 and ΔLmxM.31.2530. B-D. For deletion mutants of proteins with distal localisations: ΔLC4-like and ΔPDEB2 or ΔPDEB1. No difference between the proximal and distal flagellum were statistically significant ($p > 0.05$, two-tailed T test)
