# Supplementary material for: Proteins with proximal-distal asymmetries in axoneme localisation control flagellum beat frequency
Source: Nat Commun. 2025 Apr 4;16:3237. doi: 10.1038/s41467-025-58405-1 (PMC11971395; doi:10.1038/s41467-025-58405-1)
Supplement: Supplementary file 2 — Description of Additional Supplementary Files [file 41467_2025_58405_MOESM2_ESM.pdf]

Description of Additional Supplementary Files

Supplementary Data 1. Summary of proximal and distal proteins in T. brucei and which have orthologs in *L. mexicana.*

Supplementary Data 2. List of all tagged and/or deletion mutant cell lines generated for this study.

Supplementary Data 3. Primer sequences used for protein tagging, gene deletion and deletion validation.
