# Supplementary material for: Proteins with proximal-distal asymmetries in axoneme localisation control flagellum beat frequency
Source: Nat Commun. 2025 Apr 4;16:3237. doi: 10.1038/s41467-025-58405-1 (PMC11971395; doi:10.1038/s41467-025-58405-1)
Supplement: Supplementary file 6 — Reporting Summary [file 41467_2025_58405_MOESM6_ESM.pdf]

nature portfolio

Corresponding author(s): Richard Wheeler, Anthony Hyman

Last updated by author(s): Jan 25, 2025

Reporting Summary

Nature Portfolio wishes to improve the reproducibility of the work that we publish. This form provides structure for consistency and transparency in reporting. For further information on Nature Portfolio policies, see our Editorial Policies and the Editorial Policy Checklist.

Statistics

For all statistical analyses, confirm that the following items are present in the figure legend, table legend, main text, or Methods section.

n/a	Confirmed	
☐	☒	The exact sample size (*n*) for each experimental group/condition, given as a discrete number and unit of measurement
☐	☒	A statement on whether measurements were taken from distinct samples or whether the same sample was measured repeatedly
☐	☒	The statistical test(s) used AND whether they are one- or two-sided<br>*Only common tests should be described solely by name; describe more complex techniques in the Methods section.*
☒	☐	A description of all covariates tested
☒	☐	A description of any assumptions or corrections, such as tests of normality and adjustment for multiple comparisons
☐	☒	A full description of the statistical parameters including central tendency (e.g. means) or other basic estimates (e.g. regression coefficient) AND variation (e.g. standard deviation) or associated estimates of uncertainty (e.g. confidence intervals)
☐	☒	For null hypothesis testing, the test statistic (e.g. *F*, *t*, *r*) with confidence intervals, effect sizes, degrees of freedom and *P* value noted<br>*Give P values as exact values whenever suitable.*
☒	☐	For Bayesian analysis, information on the choice of priors and Markov chain Monte Carlo settings
☒	☐	For hierarchical and complex designs, identification of the appropriate level for tests and full reporting of outcomes
☒	☐	Estimates of effect sizes (e.g. Cohen's *d*, Pearson's *r*), indicating how they were calculated

Our web collection on statistics for biologists contains articles on many of the points above.

Software and code

Policy information about availability of computer code

Data collection	Axioimager.Z2 microscope with Hamamatsu ORCA-Flash4.0 and Andor Neo 5.5 cameras run using Zeiss Zen 2.3. EOL 210 Plus 200kV with Gatan OneView camera run using SerialEM 4.1.
Data analysis	Custom image analysis pipeline using MatLab 2024a (https://github.com/Mar5bar/flagella-analysis-pipeline), ImageJ version 1.52a.

For manuscripts utilizing custom algorithms or software that are central to the research but not yet described in published literature, software must be made available to editors and reviewers. We strongly encourage code deposition in a community repository (e.g. GitHub). See the Nature Portfolio guidelines for submitting code & software for further information.

Data

Policy information about availability of data

All manuscripts must include a data availability statement. This statement should provide the following information, where applicable:

- Accession codes, unique identifiers, or web links for publicly available datasets
- A description of any restrictions on data availability
- For clinical datasets or third party data, please ensure that the statement adheres to our policy

Data is made available through the source data file.

Research involving human participants, their data, or biological material

Policy information about studies with human participants or human data. See also policy information about sex, gender (identity/presentation), and sexual orientation and race, ethnicity and racism.

Reporting on sex and gender	N/A
Reporting on race, ethnicity, or other socially relevant groupings	N/A
Population characteristics	N/A
Recruitment	N/A
Ethics oversight	N/A

Note that full information on the approval of the study protocol must also be provided in the manuscript.

Field-specific reporting

Please select the one below that is the best fit for your research. If you are not sure, read the appropriate sections before making your selection.

☒ Life sciences ☐ Behavioural & social sciences ☐ Ecological, evolutionary & environmental sciences

For a reference copy of the document with all sections, see nature.com/documents/nr-reporting-summary-flat.pdf

Life sciences study design

All studies must disclose on these points even when the disclosure is negative.

Sample size	All cell biological experiments were designed with sample sizes based on prevailing best practice from the literature, eg. Gray et al. 2024 JCB. Sample size varies assay to assay, in practice >= 3 replicates and individually specified in the text.
Data exclusions	There was no data exclusion.
Replication	Replicability was confirmed by consistent behaviour of multiple independent replicates, as in Sample size above, in practice >= 3 replicates. Deletion mutant phenotype was qualitatively confirmed by checking motility of deletion mutants re-generated on the genetic background of tagged proteins.
Randomization	There were not experimental groups requiring randomisation. All assays involved defined reagents, ie. genetically validated clonal cell lines.
Blinding	Data analysis was, where possible, carried out single blinded to the sample identity but was not enforced. Experimental procedures were not generally blinded due to sample to sample differences in handling.

Reporting for specific materials, systems and methods

We require information from authors about some types of materials, experimental systems and methods used in many studies. Here, indicate whether each material, system or method listed is relevant to your study. If you are not sure if a list item applies to your research, read the appropriate section before selecting a response.

Materials & experimental systems

n/a	Involved in the study	
☒	☐	Antibodies
☐	☒	Eukaryotic cell lines
☒	☐	Palaeontology and archaeology
☒	☐	Animals and other organisms
☒	☐	Clinical data
☒	☐	Dual use research of concern
☒	☐	Plants

Methods

n/a	Involved in the study	
☒	☐	ChIP-seq
☒	☐	Flow cytometry
☒	☐	MRI-based neuroimaging

Eukaryotic cell lines

Policy information about cell lines and Sex and Gender in Research

Cell line source(s)	Cas9T7 L. mexicana derived from WHO strain MNHYC/BZ/62/M379 from Eva Gluenz.
Authentication	Species identity confirmed by recent genomic and RNA sequencing.
Mycoplasma contamination	Routine DNA staining is used to identify any potential mycoplasma contamination. No samples had detectable mycoplasma.
Commonly misidentified lines (See ICLAC register)	*Name any commonly misidentified cell lines used in the study and provide a rationale for their use.*

Plants

Seed stocks	None
Novel plant genotypes	None
Authentication	None
